# Functional Anatomy of the Sheep Heart as a Model for Testing Cardiovascular Devices

**DOI:** 10.17691/stm2025.17.2.02

**Published:** 2025-04-30

**Authors:** Y.L. Rusakova, I.Yu. Zhuravleva

**Affiliations:** Leading Researcher, Experimental Biological Clinic, Institute of Experimental Biology and Medicine; Meshalkin National Medical Research Center, Ministry of Health of the Russian Federation, 15 Rechkunovskaya St., Novosibirsk, 630055, Russia; Professor, Director of the Institute of Experimental Biology and Medicine; Meshalkin National Medical Research Center, Ministry of Health of the Russian Federation, 15 Rechkunovskaya St., Novosibirsk, 630055, Russia

**Keywords:** sheep heart anatomy, functional parameters of an ovine heart, sheep aortic root, experimental models, preclinical trials

## Abstract

**The aim of the study** is to assess the availability of this animal model for preclinical testing of implantable cardiovascular devices by studying numerical anatomical and functional characteristics of the ovine heart and to establish their differences from those of the human heart.

**Materials and Methods:**

The study was performed on 17 healthy crossbred Romanov sheep weighing 20–29 kg in group 1 (n=7) and 30–43 kg in group 2 (n=10). All animals underwent echocardiography examination on the Philips CX-50 apparatus (revision 3.1.2; Philips, Netherlands) with a sector-phased S5-1 sensor from the right parasternal projection (long and short axis) to determine the heart rate, right ventricular wall thickness in diastole, right and left ventricular end-diastolic dimensions (RV EDD and LV EDD), left ventricular end-systolic dimension (LV ESD), interventricular septum (IVS) thickness in systole and diastole, left ventricular posterior wall thickness in systole and diastole.

Functional parameters of the left ventricle (left ventricle end-systolic and end-diastolic volumes (LV ESV and LV EDV), ejection fraction and shortening fraction) were calculated using the modified Simpson method built into the echocardiography software. The diameter of the mitral annulus and the characteristics of the aortic root were also measured: the diameters of the aortic valve, Valsalva sinuses, and sinotubular junction, as well as the height of the aortic root from the fibrous ring to the line of the sinotubular junction. Direct measurements of the ascending aorta and pulmonary artery diameters, intercommissural distances, and the height of the aortic valve leaflets were performed after autopsy.

**Results:**

A number of anatomical and functional parameters of the sheep heart such as ejection fraction, myocardium thickness, LV EDD and LV ESD, aorta, and pulmonary artery diameters, have been established to be close to those of the human heart. At the same time, LV EDV and LV ESV of the sheep are significantly lower than in humans, even in relation to the body surface area, and the average mitral valve diameter is larger. Despite the same diameters of the aortic valve, Valsalva sinuses, and sinotubular junction, the structures of the ovine and human aortic roots are different: the sheep root features a smaller height and intercommissural distances of the cusps. In addition, some differences were found in the arrangement of the cusps in relation to the valve axis: the intercommissural distance of the right coronary leaflet was almost 2 times greater than the similar indicator of the left coronary leaflet.

Most anatomical and functional parameters have not shown any correlation with the animals’ body weight. Only in group 2, a significant positive correlation between body weight and the height of the aortic valve leaflets was found.

**Conclusion:**

The anatomical and functional characteristics of the sheep heart are close but not identical to human hearts. The sheep is a valid experimental model for preclinical testing of implantable cardiovascular devices, but a successful experiment requires careful screening of animals with echocardiographic assessment of the target zone parameters and selection of the appropriate device size.

## Introduction

The problem of developing new implantable devices for surgical treatment of acquired heart valvular diseases remains urgent due to the growth of life longevity and, respectively, increase of the population at the age over 60–70 years and older. To correct degenerative defects in elderly patients, transcatheter devices are widely used. Technical innovations and steady progress in the development of cardiosurgery also stimulate new developments of minimally invasive devices for the pediatric group of patients [1, 2].

The development of implantable medical devices in endovascular and cardiovascular surgery requires a mandatory stage of preclinical trials on large animals with orthotopic implantation of the tested device. Usually, calves [3], pigs [4], and sheep [5] are used for this purpose. The experiments with sheep are not rare in studying the technical aspects of new cardiovascular device implantation and assessing their function and long-term structural transformations [6, 7]. Primate models would be considered optimal [8] but their use would require greater financial expenses and overcoming some ethical aspects of the experiment.

When choosing the animal model and planning the experiment, it is necessary to make sure that the targeted zone of the cardiovascular system of the animal matches the implanted device in anatomical and functional parameters. Significant mismatches in sizes and characteristics will result in transplantation failure, obtaining unreliable results, or their absence. For example, obstruction of the coronary blood flow is a known problem in orthotopic transcatheter implantation of some models of self-opening aortic valves to pigs [9]. Such complications as migration of the mitral transcatheter prosthesis and/or paraprosthesis regurgitation due to noncongruence of devices developed for humans are also possible [10, 11].

Knowledge of morphometric characteristics of the intervention zone is needed in planning the trials of medical devices to diminish the risk of laboratory animal lethality and obtain valid results of the experiment. Non-effective invasive intervention and especially unnecessary death of animals are unacceptable from the bioethical point of view.

Unfortunately, we have not found today a unified protocol for studying anatomical and functional parameters of the ovine hearts in the available literature sources. As a rule, publications contained a general description of the sheep aortic root and data on the dependence of the heart size and its separate structures on the breed, gender, body mass, and age and also individual topographic features of some animals [12, 13].

**The aim of our study** is to determine numerical anatomical and functional characteristics of the ovine heart, establish their differences from the human heart, and assess the suitability of this animal model for preclinical testing of implantable cardiovascular devices.

## Materials and Methods

The study was performed on 17 healthy crossbred Romanov sheep frequently encountered in the Novosibirsk region. These animals are commonly used at the Meshalkin National Medical Research Center for preclinical testing of implantable medical devices. The sheep included in this investigation were divided into 2 groups: group 1 (n=7) consisted of animals with 20–29 kg body mass; group 2 (n=10) were the animals with 30–43 kg body mass. All sheep underwent preliminary veterinarian examination and were found to be healthy.

The study was approved by the Bioethical Committee of the Meshalkin National Medical Research Center (protocol No.3 of August 25, 2023). Housing conditions and animal handling complied with the requirements of the European Convention for the Protection of Vertebrate Animals used for Experimental and Other Scientific Purposes (Strasbourg, 2006).

Preclinical testing was carried out within the framework of the project on the development of transcatheter mitral bioprosthesis. The aortic valve was left intact, which made it possible to perform direct morphometry in all animals that died after euthanasia in the acute experiment or in the early (not exceeding 10 days) postoperative period.

**Figure 1.**
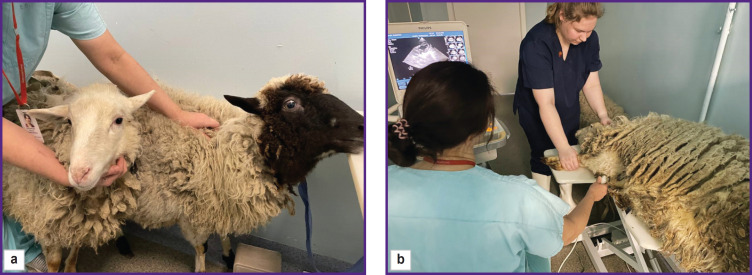
EchoCG examination of the sheep: (a) animals were brought in pairs to reduce the stress; (b) the procedure was performed in the right lateral decubitus position on the table with special cutouts for better US sensor positioning

EchoCG examination was done without sedation, therefore animals were brought in pairs and had time for adaptation before the examination in order to reduce the stress (Figure 1).

On the day of echoCG, the animals were weighed using VSP4-150 JSO scales (GK “Nevskiye vesy”, Russia), accuracy class III, GOST OIML R76-1-2011.

The right side of the chest in the region of the cardiac impulse (4–5^th^ intercostal space, the lower third) was cleared from wool using Max45 clipper (Moser, Germany). The sheep was placed on the table in the right lateral decubitus position with the thoracic limbs pulled forward holding them by the hands. The examination took about 10 min.

Philips CX-50 US system (revision 3.1.2; Philips, the Netherlands) was used for echoCG with the sector- phased S5-1 sensor in compliance with the methods previously described for animals [14–16]. Acugel of medium viscosity was applied as a contact medium to the animal skin in the area of the cardiac echo window. Images were acquired from the right parasternal projection (long and short axis). Long axis view allowed us to obtain 4-chamber and 5-chamber images.

The following parameters were registered: heart rate, right ventricular wall thickness in diastole, right and left ventricular end-diastolic dimensions (RV EDD and LV EDD), interventricular septum (IVS) thickness in systole and diastole, left ventricular posterior wall thickness in systole and diastole, left ventricular end-systolic dimension (LV ESD).

**Figure 2.**
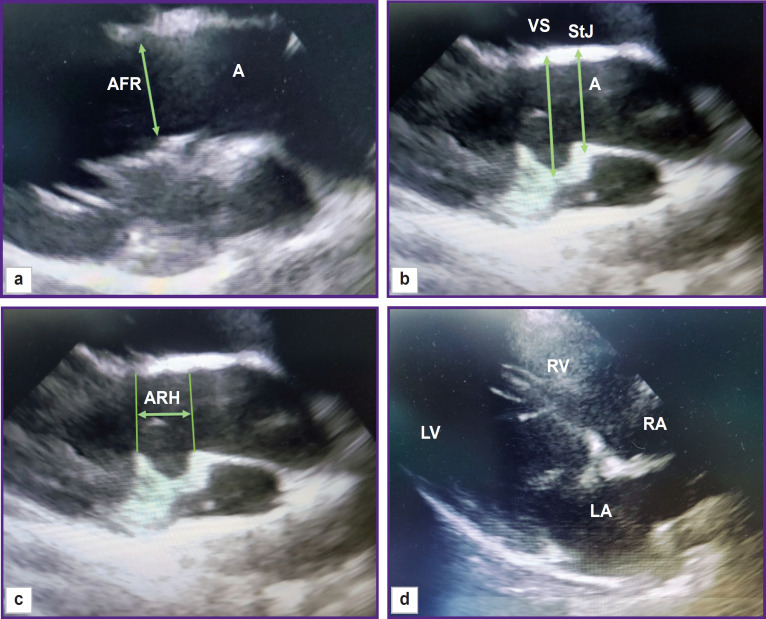
EchoCG imaging of the main ovine heart structures: (a) aorta (A), aortic fibrous ring (AFR); (b) Valsalva sinus (VS), sinotubular junction (StJ); (c) aortic root height (ARH); (d) 4-chamber position, long axis (LV — left ventricle, RV — right ventricle, RA — right atrium, LA — left atrium)

The functional parameters of the left ventricle (left ventricle end-systolic and end-diastolic volumes (LV ESV and LV EDV), ejection fraction, and shortening fraction) were calculated using the modified Simpson method built into the echocardiography software. The diameter of the fibrous aortic ring was fixed during systole at the moment of its maximal size; the cursor was placed from the inner edge to the inner edge at the point of leaflet attachments. Valsalva sinuses and the sinotubular junction were measured during diastole from the outer contour to the outer contour, the height of the aortic root — from the fibrous ring to the line of the sinotubular junction (Figure 2).

**Figure 3.**
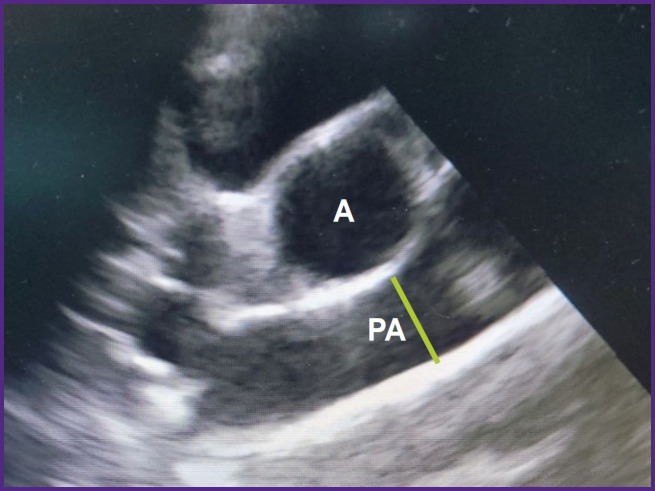
EchCG image of the pulmonary artery; right parasternal projection, shortaxis view A — aorta, PA — pulmonary artery

**Figure 4.**
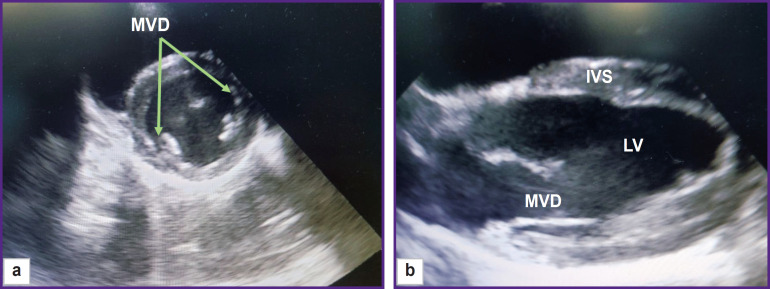
EchoCG image of the mitral valve; right parasternal projection: (a) short-axis view; (b) long-axis view; MVD — mitral valve diameter, IVS — interventricular septum, LV — left ventricle

The rotation of the sensor around its axis enabled us to obtain a short axis image of the animal heart. From this position, the pulmonary artery diameter was assessed at the valve level (Figure 3). The diameter of the mitral valve was estimated in the long- and short-axis views (Figure 4).

**Figure 5.**
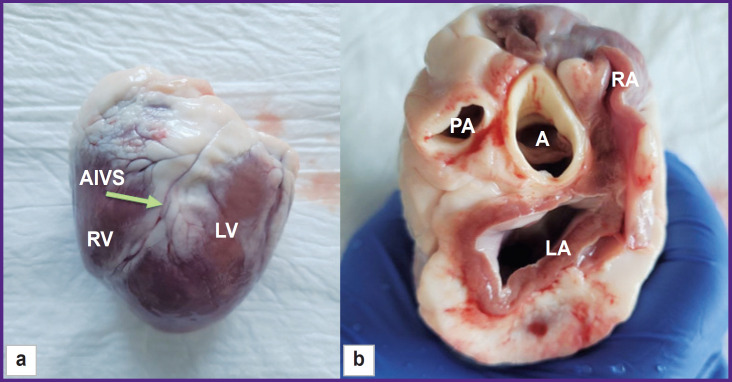
Ovine heart after explantation: (a) view from the front surface; (b) top view after the removal of atrial auricles. RV — right ventricle, LV — left ventricle, AIVS — anterior interventricular sulcus, LA — left atrium, RA — right atrium, PA — pulmonary artery, A — aorta

**Figure 6.**
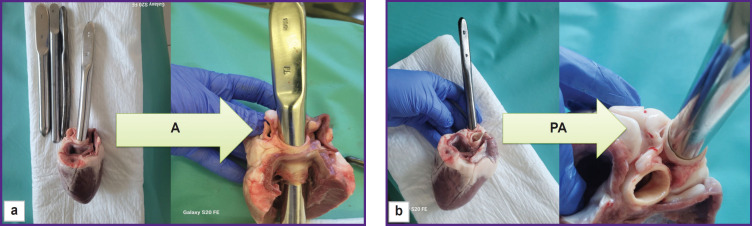
Measurement of aortic and pulmonary artery diameters using Hegar’s dilator: (a) measurement of aorta (A); (b) measurement of pulmonary artery (PA)

To make direct measurements of cardiac structures, the hearts were removed at autopsy in compliance with standard procedures (Figure 5). Using Hegar’s dilators (Figure 6), the diameter of the ascending aorta and pulmonary artery were assessed. Then, the aortic root was dissected along the commissure between the leaflets.

**Figure 7.**
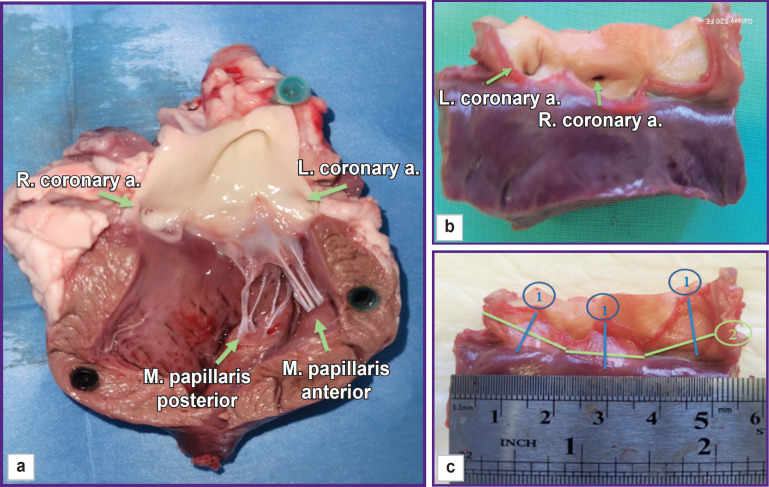
Measurement of linear parameters: (a) ovine gross heart specimen, view of opened left ventricle; (b) aortic root specimen; (c) measurement of the leaflet height (1) and intercommissural distance (2)

Measurements of linear dimensions were performed with compasses with the accuracy of 1.0 mm and the method of precise modeling of the thread across the heart structure configuration with subsequent measurement of its length with a ruler (Figure 7). Intercommissural distance was determined between the commissural tops with subsequent measurements of the length along the convex sinus wall, and a leaflet height was measured from the middle of the free leaflet margin length to the middle of the length of leaflet attachment to the fibrous ring.

Presence of any damage or abnormal structures was the criterion for exclusion from the study.

The relation of the mass to the body surface area in sheep was calculated by the formula commonly accepted in veterinary medicine [17]:

BSA=K⋅(m/2.3)⋅10−4,

where BSA is body surface area (m^2^); *m* — body mass (g); *K* — coefficient for various animal species (it is equal to 10.1 in the present study).

### Statistical data processing

Data were statistically processed using Statistica 8.0 software (StatSoft Inc., USA). The Shapiro–Wilk test was used to determine normality of distribution. Since the distribution differed from normal, methods of non-parametric statistics were employed. Data were presented as the median (Me) and interquartile interval [Q1; Q3]. The Mann–Whitney U-test was used to assess statistical significance of differences, while the Spearman’s method was applied to calculate the correlation coefficient of qualitative variables. Differences were considered statistically significant at р<0.05.

## Results and Discussion

Unfortunately, it is impossible to completely exclude the stress in animals subject to examination without sedation, therefore we observed increase of the average heart rate to 97–98 bpm, which is normally 60–80 bpm.

In all cases, investigations from the right parasternal approach in the 4–5^th^ intercostal space resulted in obtaining high-quality images, which, in turn, allowed us to make accurate measurements with the results presented in Table 1.

**T a b l e 1:** Results of echoCG examination of locally crossbred Romanov sheep, Me [Q1; Q3]

Indicator	Group 1 (n=7), 20–29 kg	Group 2 (n=10), 30–43 kg	p*
Body mass (kg)	24.0 [23.0; 25.0]	33.5 [30.0; 39.0]	0.0006
Body surface area (m^2^)	0.840 [0.817; 0.864]	1.039 [0.975; 1.142]	0.0006
Heart rate (beats/min)	98.0 [94.0; 116.0]	97.0 [96.0; 102.0]	0.7697
Right ventricular wall thickness in diastole (mm)	7.4 [6.0; 10.2]	8.7 [6.8; 9.6]	0.8073
Right ventricular end-diastolic dimension (mm)	18.0 [12.7; 33.0]	15.8 [13.8; 17.9]	0.5259
Interventricular septum thickness in diastole (mm)	9.9 [9.3; 26.4]	9.2 [8.6; 10.3]	0.2123
Left ventricular end-diastolic dimension (mm)	33.5 [24.2; 59.0]	29.9 [25.4; 38.2]	0.4349
Left ventricular posterior wall thickness in diastole (mm)	8.6 [8.3; 9.3]	9.9 [9.1; 11.4]	0.0652
Left ventricular end-diastolic volume (ml)	36.3 [20.6; 45.8]	43.8 [23.2; 62.8]	0.7630
Interventricular septum thickness in systole (mm)	11.2 [8.4; 12.4]	11.9 [10.8; 13.7]	0.3289
Left ventricular end-systolic dimension (mm)	26.8 [15.9; 30.5]	19.8 [18.6; 23.5]	0.5914
Left ventricular posterior wall thickness in systole (mm)	13.6 [10.2; 15.7]	15.5 [14.2; 15.9]	0.3291
Left ventricular end-systolic volume (ml)	7.4 [7.0; 26.6]	13.2 [11.2; 19.2]	0.7133
Ejection fraction (%)	69.2 [58.2; 79.6]	64.3 [58.0; 70.7]	0.4159
Shortening fraction (%)	38.4 [28.8; 46.8]	33.9 [29.4; 39.5]	0.4159
Diameter of aortic valve fibrous ring (mm)	18.2 [16.7; 22.9]	21.7 [20.0; 23.8]	0.1858
Diameter of Valsalva sinuses (mm)	29.2 [26.8; 30.6]	32.1 [29.5; 33.3]	0.1037
Sinotubular junction diameter (mm)	24.0 [23.5; 24.9]	24.7 [22.0; 27.0]	0.6714
Height of the Valsalva sinuses (mm)	17.6 [16.6; 26.2]	17.6 [14.9; 18.5]	0.4989
Ascending aorta diameter (mm)	23.4 [22.6; 24.2]	24.2 [22.9; 24.7]	0.3579
Pulmonary artery diameter (mm)	21.6 [16.9; 25.0]	20.5 [17.3; 23.6]	0.8708
Mitral valve diameter (mm)	31.0 [30.3; 35.7]	33.9 [31.2; 36.8]	0.4477

* statistical significance of value differences between the indicators of group 1 and 2.

The data obtained by direct measurement of the aorta and pulmonary artery diameters (see Figure 6) agreed with those acquired by the echoCG examination (Table 2). Thus, when planning experimental operation, dimensions of the implanted device may be chosen with high accuracy based on the results of the echoCG examination.

**T a b l e 2:** Diameters of the ascending aorta and pulmonary artery according to the echoCG data and direct measurement, Me [Q1; Q3]

Indicator	Group 1 (n=7), 20–29 kg		Group 2 (n=10), 30–43 kg	
	EchoCG	Direct measurement	EchoCG	Direct measurement
Ascending aorta diameter (mm)	23.4 [22.6; 24.2]	24.0 [23.0; 24.0]	24.2 [22.9; 24.7]	24.0 [23.0; 25.0]
Pulmonary artery diameter (mm)	21.6 [16.9; 25.0]	21.5 [17.0; 25.0]	20.5 [17.3; 23.6]	20.5 [17.0; 24.0]

The results obtained in the present study have demonstrated a high individual variability of anatomical and functional parameters of ovine hearts. Moreover, none of the parameters given in Table 1 showed a correlation with the animal body mass (the correlation for all indicators was assessed as weak at ρ<0.4). In animals from group 2, only statistically insignificant tendency to the increase of some measured structures was noted: ventricular wall thickness, intraventricular septum, ventricle sizes, aorta from the fibrous ring to its ascending part, and the mitral valve.

Notable that some anatomical and functional indicators of the ovine heart are very similar to the human heart. So, ejection fraction in sheep is 58–79%, which is close to the human reference values (53– 77%) [18, 19]. The posterior wall thickness of the left ventricle in humans is 8–11 mm, in sheep, it is 8.6– 9.9 mm, the TVS in diastole is 9–12 and 9.2–9.9 mm, respectively [20].

Despite the fact that LV ESD and LV EDD in sheep is smaller on average than in human (human ESD is equal to 21.6–39.8 mm, EDD — 37.8–58.4 mm [18]), one can easily find animals in group 1 and 2, whose indicators are within the reference values for healthy people. The LV ESD and LV EDD in sheep are highly variable, especially in animals of group 1, and are in the range of 15.9–30.5 and 24.4–59.0 mm, respectively.

At the same time, the volume values of the ovine left ventricle are much lower than the human ones even in relation to the body surface area (Table 3).

**T a b l e 3:** Values of left ventricular end-systolic volume (LV ESV) and left ventricular end-diastolic volume (LV EDV) in humans and sheep, Me [Q1; Q3]

Indicator	Sheep		Women, 20–80 years old [18]
	Group 1 (n=7), 20–29 kg	Group 2 (n=10), 30–43 kg	
LV EDV (ml)	36.3 [20.6; 45.8]	43.8 [23.3; 62.8]	86–178
LV EDV/BSA (ml/m^2^)	43.2 [25.2; 53.0]	42.2 [22.3; 55.0]	56–96
LV ESV (ml)	7.4 [7.0; 26.6]	13.2 [11.2; 19.2]	22–66
LV ESV/BSA (ml/m^2^)	8.8 [8.6; 30.8]	12.7 [11.5; 16.8]	14–34

N o t e. BSA is body surface area.

The diameter of the mitral valve is a bit greater than in humans (31–34 mm): the average dimensions are 29 mm in men and 26 mm in women [21]. The diameters of the ascending aorta and pulmonary artery (see Table 1 and 2) in sheep approximate to those of human (19–35 and 16–29 mm, respectively) [22].

Of great interest for the *in vivo* testing of the aortic valve prostheses is the geometry of the ovine aortic root, the compliance of its dimensions with the human ones. The fibrous ring diameter of the aortic valve in men is known to be in the range of 16–23 mm [19]. In the sheep of our study, these values were 16.7 mm in group 1 up to 23.8 mm in group 2 (see Table 1). The diameters of Valsalva sinuses and sinotubular junction in sheep are quite comparable with similar human indicators, but their variability is somewhat less. Thus, the diameter of Valsalva sinuses in sheep is 26.8– 33.3 mm, while in men it is 22–35 mm, the sinotubular junction diameter is 23.5–27.0 and 18–30 mm, respectively [19].

**Figure 8.**
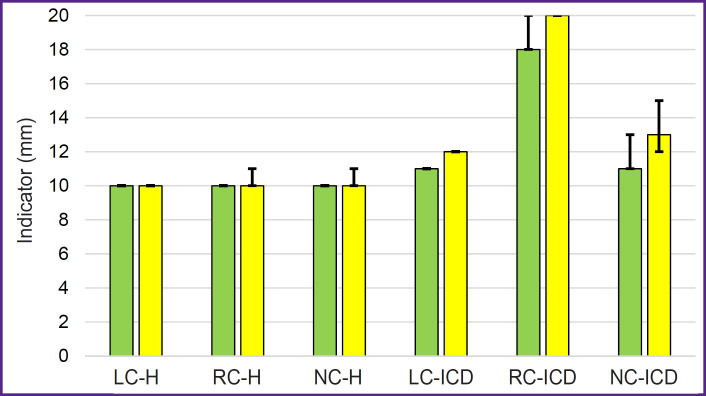
Results of measuring aortic valve leaflets in sheep weighing 20– 29 kg (*green color*) and 30–43 kg (*yellow color*) LC-H — left coronary leaflet height, RC-H — right coronary leaflet height, NC-H — non-coronary leaflet height; LC-ICD — intercommissural distance of the left coronary leaflet, RC-ICD — intercommissural distance of the right coronary leaflet, NC-ICD — intercommissural distance of the noncoronary leaflet

The results of direct measurements have shown that the leaflet height of the aortic valve is about 10–12 mm regardless of the animal mass (Figure 8). It is about twice as much in humans and is equal to 19–24 mm [23]. Still greater differences have been found in the location of leaflet commissures relative to the valve axis. The distance between the leaflet commissures of the human aortic valve is rather uniform and is 24–33 mm [23], whereas those measured by us in sheep amounted to 11–13 mm for the left coronary leaflet and 11–15 mm for the non-coronary leaflet.

In sheep of group 2, the intercommissural distances were 1–2 mm larger (see Figure 8) than in sheep of group 1, although these differences were not statistically significant (р>0.1). The inercommissural distance of the right coronary leaflet was 18–20 mm in both groups. A positive Spearman correlation has been found between the body mass and this commissural distance (ρ=0.82 at р<0.05) in animals of group 1. In animals of group 2, a positive Spearman correlation was detected between the body mass and intercommissural distance of the left coronary leaflet (ρ=0.82 at р<0.05), between the body mass and the height of the left coronary leaflet (ρ=0.72 at р<0.05), body mass and the height of the right coronary leaflet (ρ=0.85 at р<0.05), body mass and the height of the non-coronary leaflet (ρ=0.83 at р<0.05).

The conducted investigations made us come to the conclusion that anatomical and functional characteristics of the ovine heart are close but not identical to the human ones. These characteristics in men are directly connected with the body mass, age, height, body surface area, and so on [24, 25]. However, there are no such correlations for the ovine hearts, therefore, the choice of animals for a specific experiment should not be based on their mass. For example, the aortic valve with 23 mm in diameter or a mitral valve with a diameter of 35 mm may be equally often encountered in the group with an average mass of 24 kg and in the animals weighing 10 kg more. At the same time, it should be noted that we have not found any significant correlations between the diameters of these valves and indicators of linear and volumetric dimensions of the heart. But correlations have been found between the animal mass and aortic root structure, which should be taken into account in selecting the object of the experiment.

Smaller volumetric dimensions of ovine cardiac cavities relative to the human heart also should be taken into consideration. It is especially important for testing transcatheter prostheses of the mitral valve, which are designed for patients with ischemic mitral regurgitation and, consequently, a “large” left ventricle.

We consider preliminary modeling *in silico* to be an optimal approach to planning the experiment: successive echoCG screening of animals, 3D modeling of the targeted zone with subsequent virtual implantation of the tested device, selection of the animal with optimal anatomo-functional characteristics and the size of the implanted device — and only after this a direct surgical experiment may be fulfilled.

The results of our study allowed us to outline several practical aspects, which should be considered when you analyze the characteristics of the targeted zone.

First, echoCG examination in sheep without sedation is complicated by sensitivity of these animals to stress with the development of tachypnea and tachycardia. However, sedation requires preparation of animals (at least food deprivation for 12 h), and also provokes the decrease of systolic and diastolic function during anesthesia. Besides, general anesthesia in sheep reduces heart contractility [26]. We consider nonmedicamentous measures of stress reduction to be more useful: extremely careful handling, examination in pairs, manual recording, treating the sheep with mineral salt lick during the procedure.

Second, difficulties of acquiring high quality images caused by the keeled chest, narrow intercostal spaces, and presence of gas in the sheep rumen should be regarded as well [3]. We also ran into this problem but succeeded in obtaining the necessary data since all echoCG examination procedures were performed from the right lateral decubitus position using parasternal long-axis and short-axis views.

Preparing for the experiment on aortic valve implantation, one should consider two factors discovered by us in the process of our investigation. The first one consists in the direct correlation between the animal body mass and leaflet height and also between the mass and commissural distance. The second factor refers to great differences in commissural distances. The human aortic valve is more axisymmetric and demonstrates significant correlations between the key leaflet parameters [27]. These factors should be taken into account since some models of implanted valves may cause coronary obstruction if anatomical parameters of the targeted zone are underestimated.

Thus, the results obtained have shown the necessity of mandatory echocardiographic examination of the targeted zone prior to the execution of the surgical experiment.

## Conclusion

The presented numerical anatomical and functional characteristics of healthy crossbred Romanov sheep hearts have demonstrated their differences from the human heart parameters, which does not prevent from recognizing these sheep a valid model for preclinical trials of implanted cardiovascular devices.

The obtained data will be useful for cardiologists, surgeons, and engineers engaged in the development of devices for surgery of mitral, aortic, pulmonary artery valves and their preclinical testing on animals.

## References

[1] Blaser M.C. Kraler S. Lüscher T.F. Aikawa E. Multi- omics approaches to define calcific aortic valve disease pathogenesis. Circ Res 2021; 128(9):1371–1397, 10.1161/CIRCRESAHA.120.317979. 33914608 PMC8095729

[2] Bouma B.J. Mulder B.J. Changing landscape of congenital heart disease. Circ Res 2017; 120(6):908–922, 10.1161/CIRCRESAHA.116.309302. 28302739

[3] Karimov J.H. Moazami N. Kobayashi M. Sale S. Such K. Byram N. Sunagawa G. Horvath D. Gao S. Kuban B. Golding L.A. Fukamachi K. First report of 90- day support of 2 calves with a continuous-flow total artificial heart. J Thorac Cardiovasc Surg 2015; 150(3):687–693.e1, 10.1016/j.jtcvs.2015.06.023. 26173607 PMC4554829

[4] Rusakova Y.L. Grankin D.S. Podolskaya K.S. Zhuravleva I.Y. Pigs as models to test cardiovascular devices. Biomedicines 2024; 12(6): 1245, 10.3390/biomedicines12061245. 38927452 PMC11200718

[5] Barbarash L.S. Klyshnikov K.Yu. Haes B.L. Halivopulo I.K. Stasev A.N. Krutitsky S.S. Borisenko D.V. Sitnikov M.A. Ivanova A.V. Kudryavtsev Yu.A. Kokorin S.G. Evtushenko A.V. Ovcharenko E.A. First experience of sutureless redo on mitral valve using “valvein-valve” method: two-stage implantation on a large animal. Byulleten’ eksperimental’noy biologii i meditsiny 2019; 168(12):783–787. 10.1007/s10517-020-04809-6 32328943

[6] Kim D.H. Morris B. Guerrero J.L. Sullivan S.M. Hung J. Levine R.A. Ovine model of ischemic mitral regurgitation. Methods Mol Biol 2018; 1816: 295–308, 10.1007/978-1-4939-8597-5_23. 29987829 PMC6207369

[7] Van Hoof L. Claus P. Jones E.A.V. Meuris B. Famaey N. Verbrugghe P. Rega F. Back to the root: a large animal model of the Ross procedure. Ann Cardiothorac Surg 2021; 10(4):444–453, 10.21037/acs-2020-rp-21. 34422556 PMC8339627

[8] Sampath S. Klimas M. Feng D. Baumgartner R. Manigbas E. Liang A.L. Evelhoch J.L. Chin C.L. Characterization of regional left ventricular function in nonhuman primates using magnetic resonance imaging biomarkers: a test-retest repeatability and inter-subject variability study. PLoS One 2015; 10(5): e0127947, 10.1371/journal.pone.0127947. 26010607 PMC4444127

[9] Cai J. Huang H. Zhou Y. Mei Y. Shao J. Wang Y. A new type of aortic valved stent with good stability and no influence on coronary artery. J Cardiothorac Surg 2013; 8: 210, 10.1186/1749-8090-8-210. 24219844 PMC3842839

[10] Joudinaud T.M. Flecher E.M. Curry J.W. Kegel C.L. Weber P.A. Duran C.M. Sutureless stented aortic valve implantation under direct vision: lessons from a negative experience in sheep. J Card Surg 2007; 22(1):13–17, 10.1111/j.1540-8191.2007.00337.x. 17239204

[11] Bai Y. Zong G.J. Wang Y.Y. Jiang H.B. Li W.P. Wu H. Zhao X.X. Qin Y.W. Percutaneous aortic valve replacement using a W-model valved stent: a preliminary feasibility study in sheep. Chin Med J (Engl) 2009; 122(6):655–658. 19323929

[12] Acorda J.A. Pajas A.M.G. M-mode echocardiographic values in male and female Philippine sheep (Ovis aries) (Artiodactyla: Bovidae) by age and status of lactation and pregnancy. Philipp J Vet Med 2015; 52(1):11–20.

[13] Hong T. Maish M.S. Cohen J. Fitzpatrick P. Bert A.A. Harper J.S. 3rd Fang D. Hoffman-Kim D. Hopkins R.A. Reproducible echocardiography in juvenile sheep and its application in the evaluation of a pulmonary valve homograft implant. Contemp Top Lab Anim Sci 2000; 39(5):20–25. 11040870

[14] Vloumidi E.I. Fthenakis G.C. Ultrasonographic examination of the heart in sheep. Small Ruminant Research 2017; 152: 119–127, 10.1016/j.smallrumres.2016.12.019.

[15] Poser H. Semplicini L. De Benedictis G.M. Gerardi G. Contiero B. Maschietto N. Valerio E. Milanesi O. Semplicini A. Bernardini D. Two-dimensional, M-mode and Doppler-derived echocardiographic parameters in sedated healthy growing female sheep. Lab Anim 2013; 47(3):194–202, 10.1177/0023677213486895. 23760962

[16] Boon J.A. Two-dimensional and M-mode echocardiography for the small animal practitioner. Ames, Iowa: John Wiley & Sons Inc; 2017.

[17] Onkologiya melkikh domashnikh zhivotnykh [Oncology of small domestic animals]. Pod red. Trofimtsova D.V., Vilkovyskogo I.F. [Trofimtsov D.V. Vilkovyskiy I.F. (editors)]. Moscow: Izdatel’skiy dom “Nauchnaya biblioteka”; 2017.

[18] Lang R.M. Badano L.P. Mor-Avi V. Afilalo J. Armstrong A. Ernande L. Flachskampf F.A. Foster E. Goldstein S.A. Kuznetsova T. Lancellotti P. Muraru D. Picard M.H. Rietzschel E.R. Rudski L. Spencer K.T. Tsang W. Voigt J.U. Recommendations for cardiac chamber quantification by echocardiography in adults: an update from the American Society of Echocardiography and the European Association of Cardiovascular Imaging. J Am Soc Echocardiogr 2015; 28(1):1–39.e14, 10.1016/j.echo.2014.10.003. 25559473

[19] Petersen S.E. Khanji M.Y. Plein S. Lancellotti P. Bucciarelli-Ducci C. European Association of Cardiovascular Imaging expert consensus paper: a comprehensive review of cardiovascular magnetic resonance normal values of cardiac chamber size and aortic root in adults and recommendations for grading severity. Eur Heart J Cardiovasc Imaging 2019; 20(12):1321–1331, 10.1093/ehjci/jez232. 31544926

[20] Vandroux D. Houehanou Y.C. Magne J. Saka D. Sonou A. Amidou S. Houinato D. Preux P.M. Aboyans V. Lacroix P. Normal reference values of cardiac chamber sizes and functional parameters in a beninese community population: the TAHES study. Int J Cardiovasc Imaging 2023; 39(9):1729–1739, 10.1007/s10554-023-02892-0. 37354384

[21] Ricci F. Aung N. Gallina S. Zemrak F. Fung K. Bisaccia G. Paiva J.M. Khanji M.Y. Mantini C. Palermi S. Lee A.M. Piechnik S.K. Neubauer S. Petersen S.E. Cardiovascular magnetic resonance reference values of mitral and tricuspid annular dimensions: the UK Biobank cohort. J Cardiovasc Magn Reson 2020; 23(1): 5, 10.1186/s12968-020-00688-y. 33407573 PMC7788733

[22] Beck L. Mohamed A.A. Strugnell W.E. Bartlett H. Rodriguez V. Hamilton-Craig C. Slaughter R.E. MRI measurements of the thoracic aorta and pulmonary artery. J Med Imaging Radiat Oncol 2018; 62(1):64–71, 10.1111/1754-9485.12637. 28762641

[23] Matsushima S. Karliova I. Gauer S. Miyahara S. Schäfers H.J. Geometry of cusp and root determines aortic valve function. Indian J Thorac Cardiovasc Surg 2020; 36(Suppl 1): 64–70, 10.1007/s12055-019-00813-2. PMC7525730 33061186

[24] Svedenhag J. Larsson T.P. Lindqvist P. Olsson A. Rythén Alder E. Individual reference values for 2D echocardiographic measurements. The Stockholm — Umeå Study. Clin Physiol Funct Imaging 2015; 35(4):275–282, 10.1111/cpf.12161. 24810718

[25] Wenzel J.P. Petersen E. Nikorowitsch J. Senftinger J. Sinning C. Theissen M. Petersen J. Reichenspurner H. Girdauskas E. Transthoracic echocardiographic reference values of the aortic root: results from the Hamburg City Health Study. Int J Cardiovasc Imaging 2021; 37(12):3513–3524, 10.1007/s10554-021-02354-5. 34324091 PMC8604854

[26] Jazwiec T. Malinowski M. Proudfoot A.G. Eberhart L. Langholz D. Schubert H. Wodarek J. Timek T.A. Tricuspid valvular dynamics and 3-dimensional geometry in awake and anesthetized sheep. J Thorac Cardiovasc Surg 2018; 156(4):1503–1511, 10.1016/j.jtcvs.2018.04.065. 29804662

[27] Odinokova S.N. Nikolenko V.N. Komarov R.N. Vinokurov I.A. Mnatsakanyan G.V. Belkharoeva R.Kh. The correlations of morphometric parameters of structures of the aortic root having practical significance in the surgical correction of the aortic valve. Morphological newsletter 2020; 28(1):30–36, 10.20340/mv-mn.2020.28(1):30-36.

